# Insights into the Mechanism Underlying the Influence of Glycation with Different Saccharides and Temperatures on the IgG/IgE Binding Ability, Immunodetection, In Vitro Digestibility of Shrimp (*Litopenaeus vannamei*) Tropomyosin

**DOI:** 10.3390/foods12163049

**Published:** 2023-08-14

**Authors:** Jinlong Zhao, Jin Wang, Lili Xu, Hao Wang, Ziye Zhang, Hong Lin, Zhenxing Li

**Affiliations:** 1 College of Food Science and Engineering, Ocean University of China, No. 5, Yushan Road, Qingdao 266003, China; kinlongzhao@163.com (J.Z.); wanghao@ouc.edu.cn (H.W.); zhangziye@ouc.edu.cn (Z.Z.); linhong@ouc.edu.cn (H.L.); 2 Key Laboratory of Environmental Medicine and Engineering, Ministry of Education, and Department of Nutrition and Food Hygiene, School of Public Health, Southeast University, Nanjing 210009, China; 3 Institute of Agro-Food Science and Technology, Shandong Academy of Agricultural Sciences, No. 202 Gongye North Road, Jinan 250100, China; xulili8339@163.com

**Keywords:** shrimp (*Litopenaeus vannamei*), tropomyosin, glycation modification, IgG/IgE binding ability, immunodetection, in vitro digestibility, structure changes

## Abstract

Tropomyosin (TM) is a heat-stable protein that plays a crucial role as a major pan-allergen in crustacean shellfish. Despite the high thermal stability of the TM structure, its IgG/IgE binding ability, immunodetection, and in vitro digestibility can be negatively influenced by glycation during food processing, and the underlying mechanism remains unclear. In this study, TM was subjected to glycosylation using various sugars and temperatures. The resulting effects on IgG/IgE-binding capacity, immunodetection, and in vitro digestibility were analyzed, meanwhile, the structural alterations and modifications using spectroscopic and LC-MS/MS analysis were determined. Obtained results suggested that the IgG/IgE binding capacity of glycosylated TM, immunodetection recovery, and in vitro digestibility were significantly reduced depending on the degree of glycosylation, with the greatest reduction occurring in Rib-TM. These changes may be attributable to structural alterations and modifications that occur during glycosylation processing, which could mask or shield antigenic epitopes of TM (E3: 61–81, E5b: 142–162, and E5c: 157–183), subsequently reducing the immunodetection recognition and digestive enzyme degradation. Overall, these findings shed light on the detrimental impact of glycation on TMs potential allergenicity and digestibility immunodetection and provide insights into the structural changes and modifications induced by thermal processing.

## 1. Introduction

Shrimp is a vital primary source of protein to sustain the growing global population, owing to its abundance of high-quality proteins and nutrients [1,2]. Annually, over 900,000 tons of shrimp are utilized as raw material in various processed foods, such as shrimp seasoning, shrimp paste, fried snack foods, and more [1]. However, despite its benefits, even a small amount of shrimp can trigger severe allergic reactions that endure for life. The increasing consumption of shrimp has led to a significant rise in the prevalence of this allergenic problem, affecting up to 2.5% of the general population globally [1,2].

Despite the high prevalence of crustacean allergies, no effective therapy is currently available, leaving diet avoidance as the only recommended course of action. However, this poses a challenge for allergic consumers, as crustacean ingredients are commonly used in a wide range of food products [2,3]. Therefore, several highly sensitive methods have been developed to detect crustacean residues, including enzyme-linked immunosorbent assay (ELISA), polymerase chain reaction (PCR) assay, and liquid chromatography-mass spectrometry (LC-MS) [1,4,5]. Among these techniques, ELISA is the most widely used method for allergen quantification due to its simplicity, high throughput detection capabilities, low cost, and high sensitivity and specificity. However, ELISA has limitations in detecting denatured proteins, as it tends to yield poorer recovery compared with native protein targets. This is because denatured proteins undergo structural changes during processing, which can affect their immunorecognition in the ELISA assay [1].

In the thermal processing of food, in addition to generating single heat-induced protein structural changes, there are also interactions and modifications between the food matrix components and proteins, with the most important modification being glycation reactions between carbohydrates and proteins. Glycation reactions are widely present in the thermal processing of food and are one of the common interactions between proteins and sugars, also known as the Maillard reaction. In the process of glycation reactions, covalent bonds are formed between the free amino groups of proteins, primarily lysine (Lys, K) and arginine (Arg, R), and the carbonyl groups of reducing sugars through the amino acids of proteins [6,7].

Tropomyosin (TM) is a significant allergen that has been found in different crustaceans. It possesses high heat stability attributed to its distinctive α-helical coiled-coil structure, making it responsible for triggering a positive IgE-mediated allergic response in 72–98% of individuals with shrimp allergies. Significantly, TM in crustacean seafood is a type of protein rich in Lysine (Lys) that is prone to undergo glycation reactions with reducing sugars during the thermal processing of food. Researchers, both domestically and internationally, have extensively utilized glycation treatment on crustacean seafood TM to investigate its structural changes and allergenicity reduction. However, research findings have shown that when different sugar molecules are used to glycate TM, the allergenicity of TM can vary to different degrees (increase, decrease, or remain unchanged). Hence, it can be inferred that glycation reactions can alter the structure of TM and lead to the destruction, masking, exposure, or generation of new antigenic epitopes, resulting in varying degrees of change in allergenicity [6,8,9,10]. However, little attention has been paid by researchers both domestically and internationally to the influence of glycation treatment on the immunodetection of target allergens. Studies have found that immunodetection frequently exhibits significant decreases in recovery rates and even false negatives in the detection of allergens in thermally processed crustacean foods [1,11,12]. However, the molecular mechanisms underlying the effect of glycation treatment on the recovery rates of target allergen immunodetection in thermally processed food are still unclear.

Therefore, the present study aims to further investigate the effects of glycation treatment using different types of sugar molecules and different temperatures on shrimp TM, including its IgG/IgE binding capacity, immunodetection, in vitro digestibility, and structure. Additionally, the glycation modification sites will be further elucidated through HPLC-MS/MS analysis to clarify the molecular mechanisms underlying the reduction in immunodetection recovery of shrimp TM caused by various glycation treatments.

## 2. Materials and Methods

### 2.1. Materials

Purified raw TM with at least 95% purity was obtained, as described in our recent study [13]. Porcine pepsin, trypsin, and 8-Anilino-1-naphthalenesulfonate (ANS) were acquired from Sigma-Aldrich (St. Louis, MO, USA). Ovalbumin (OVA), bovine serum albumin (BSA), Tween 20, and various sugars [glucose (180.16 Da), ribose 150.13 Da, lactose (342.3 Da), trehalose (342.297 Da), chitosan oligosaccharide (average 5000 Da)] acquired from Beijing Solarbio Science & Technology Co., Ltd. (Beijing, China). Rat and rabbit anti-shrimp TM IgG, anti-shrimp TM monoclonal antibody (mAb) were obtained as described in our previous study [5], with titers of 2.56 × 10^6^, 1.28 × 10^6^, and 4.096 × 10^6^ in rat antisera, rabbit antisera, and mAb, respectively. Horseradish peroxidase (HRP) labeled rabbit-anti-rat and goat-anti-rabbit IgG were purchased from Bai Aotong Experimental Materials Center (Luoyang, Henan Province, China). Horseradish peroxidase (HRP) conjugated goat anti-human IgE, and goat anti-rabbit IgG was purchased from Zhongshan Jinqiao Biotechnology Co., Ltd. (Beijing, China). All chemicals used in the study were of analytical grade.

### 2.2. Shrimp Allergic Patient Sera

All clinically shrimp-allergenic patient sera (n = 6) and 2 normal human sera were collected from the Affiliated Hospital of Qingdao University (Qingdao, China) and tested using an ImmunoCAP system (Phadia AB, Uppsala, Sweden) (Table S1). Written informed consent was required from each patient, and the study was approved by the ethics committee of the Affiliated Hospital of Qingdao University (File No. QD-PF-20210325026). All positive sera (n = 6, >0.35 UA/mL) were mixed as the positive pooled sera. Sera from two normal subjects (n = 2, <0.35 UA/mL) without an allergic history were employed as negative controls.

### 2.3. Preparation of Glycated TM

Preparations of glycosylated samples of shrimp TM with different sugars at the same temperature and with the same sugar at different temperatures were prepared with slight modifications to the method described by Zhang et al. [14] Glucose (Glu), ribose (Rib), lactose (Lac), trehalose (Tre), and chitosan (Chi) were individually mixed with shrimp TM at a ratio of 1:3 (*w*/*w*) in a glycosylation sample buffer (1 mM NaHCO_3_, 5 mM DTT) at a concentration of 2 mg/mL. The mixtures were thoroughly mixed and filled into 2 mL centrifuge tubes, which were subjected to vacuum freeze-drying to obtain different sugar–TM mixtures. Subsequently, the dried samples were subjected to glycosylation in a desiccator containing anhydrous magnesium nitrate. For the preparation of glycosylated samples with the same sugars at different temperatures, the samples were subjected to glycosylation at 60 °C for 8 h. For the preparation of glycosylated samples with the same sugar (Rib) at different temperatures, Rib was used as the research target, and the samples were separately subjected to glycosylation at 25 °C, 40 °C, 50 °C, 60 °C, 70 °C, 80 °C, and 100 °C for 8 h. The control groups consisted of samples without added sugar and samples treated with heat. After the glycosylation reaction, the samples were dissolved in ultrapure water and dialyzed using PBS (3 kDa) to remove unreacted sugar components. Subsequently, the dialyzed glycosylated samples were placed into 2 mL centrifuge tubes and subjected to vacuum freeze-drying to obtain different sugar–TMs and different temperature–TM glycosylated samples, which were stored at −20 °C for future use.

### 2.4. SDS-PAGE Analysis

The glycosylated samples were separated using gel electrophoresis using 5% stacking gels and 15% resolving gel, as described by Zhao et al. [15]. After mixing and boiling the samples with the 4 × loading buffer, different glycosylated TMs (2 μg) were added to each gel lane and electrophoresed at 80 V for 30 min at 120 V for 1 h. These gels were stained with a solution of Coomassie Brilliant Blue stain (0.25% Coomassie Brilliant Blue R-250, 45% methanol, and 45% acetic acid) for 10 min. After staining, the gel was destained until the protein bands were clearly visible, and then it was photographed using a BIO RAD Uni-versal Hood II gel imaging system (Bio-Rad Laboratories, Inc., Hercules, CA, USA).

### 2.5. Glycation Degree Determination

The free amino group content of each glycosylated sample was determined using the o-Phthalaldehyde (OPA) method [16]. For each sample, 200 μL of solution at a concentration of 0.2 mg/mL was mixed with 4 mL of OPA solution and allowed to react for 2 min at 25 °C in a light-protected environment. The absorbance of these samples was measured at a wavelength of 340 nm using a UV spectrophotometer, with L-serine serving as the standard.

### 2.6. Western Blotting (WB) Analysis

Immunoblotting was performed following the method described by Zhao et al. [13]. In brief, the glycosylated samples were resolved on a separating gel, which was then transferred onto a PVDF membrane using iBlot^TM^ gel transfer stacks (Thermo Fisher Scientific, MA, USA). After blocking with 1% (*w*/*v*) BSA, the membrane was incubated with a diluted solution of rat anti-TM sera (1:10,000 in PBST), rabbit anti-TM sera (1:20,000 in PBST), and TM monoclonal antibodies (1:5000 in PBST) for 2 h in an incubator. For IgE immunoblotting, the membrane was reacted with pooled shrimp patients’ sera (diluted 1:4) overnight at 4 °C using an incubator. Subsequently, HRP-labeled goat anti-rabbit IgG (1:10,000) and HRP-labeled goat anti-human IgE (1:2000) were added at room temperature for a 1 h incubation. After washing the membrane three times with PBST (10 mM PBS, 0.15 M NaCl, 0.05% Tween 20, pH 7.4) for 10 min each, the results were analyzed using a BIO RAD Universal Hood II gel imaging system (Bio-Rad Laboratories, Inc., CA, USA) with multiple exposures and ECL solutions to obtain an optimal signal.

### 2.7. Indirect ELISA Analysis

In indirect ELISA, the glycosylated samples were diluted using 50 mM Na_2_CO_3_-NaHCO_3_ (CBS) buffer (pH 9.6) to 5 μg/mL and coated in a Corning-Costar ELISA microplate (Corning, NY, USA) with 100 μL per well at 4 °C overnight. Following this, the plates were blocked with 1% (*w*/*v*) BSA (200 μL/well) at 37 °C for 2 h. These plates were then incubated with rat antisera (diluted 200,000), rabbit antisera (diluted 320,000), and TM monoclonal antibodies (diluted 1:20,000) against TM for 1 h. In addition, polled shrimp-allergic patients’ sera diluted 10 with PBST at 37 °C for 1.5 h diluted 10-fold with PBST were added and incubated at 37 °C for 1.5 h. These plates were subsequently washed and incubated with HRP-labeled rabbit anti-rat IgG (100 μL, diluted 1:10,000), HRP-labeled goat anti-rabbit IgG (100 μL, diluted 1:10,000), HRP-labeled rabbit anti-mouse IgG (100 μL, diluted to 1:10,000) or HRP-labeled goat anti-human IgE (100 μL, diluted 1:3000) for 1 h at 37 °C. Afterward, the enzyme reaction was initiated upon adding the TMB solution and stopped after adding sulfuric acid. The absorbance was measured at 450 nm using a MultiscanMK3 microplate reader (Thermo Fisher Scientific Inc., Waltham, MA, USA).

### 2.8. Immunodetection Recovery Determination

The immunodetection recovery of the glycosylated samples was determined using PcAb and mAb sELISA analysis following the method previously reported by Zhao et al. [5]. To summarize, the experimental procedure involved the following steps: First, plates were coated with rat anti-TM sera (diluted 1:100,000) and anti-shrimp TM mAb (diluted 1:10,000), which was diluted with CBS. The plates were then blocked using a 1% (*w*/*v*) BSA solution, as previously described. Next, the assay was conducted by adding TM standards or differently treated samples, all at an equal concentration (100 μL/well). The plates were incubated for 20 min at 37 °C. Afterward, the wells were washed three times with PBST (10 mM PBS, 0.15 M NaCl, 0.05% Tween 20, pH 7.4). Subsequently, 100 μL/well of rabbit anti-TM sera, diluted 1:10,000 with PBST, were added and incubated for 60 min at 37 °C. Following another round of washing, the plates were incubated with HRP-labeled anti-TM antibodies, diluted 1:10,000 in PBST, for 60 min at 37 °C. Finally, the subsequent determination was performed according to the aforementioned protocol.

### 2.9. In Vitro Digestion Assay

In vitro simulated gastrointestinal digestion of the glycosylated samples was performed as previously described by Zhao et al. [15]. During the simulated gastric digestion process, various glycosylated samples were combined with equal volumes of simulated gastric fluids (SGF) containing pepsin (200 U/mL of digestate). The mixture was then incubated at 37 °C with constant stirring. At specific time intervals (0, 5, 10, 20, 30, 60, and 120 min), 100 μL aliquots of the digested samples were transferred to centrifuge tubes. To stop the digestion process, a Na_2_CO_3_ solution (30 μL, 0.2 M) was immediately added. Similarly, in the simulated intestinal digestion process, glycosylated samples were mixed with equal volumes of simulated intestinal fluids (SIF) containing trypsin (100 U/mL of digestate). The mixture was incubated for the same time intervals as in the gastric digestion process. After each time interval, aliquots of the digested solution were taken out, and the digestion process was halted by adding Pefabloc^®^ (5 mM final concentration) for further analysis. Finally, all the digestion samples were subjected to SDS-PAGE analysis.

### 2.10. CD Spectral Analysis

CD spectrum data of the glycosylated samples between 190 and 260 nm were collected using a Jasco-810 spectropolarimeter (JASCO, Japan Spectroscopic Co. Inc., Tokyo, Japan) at 25 °C. The samples, each at a concentration of 0.2 mg/mL, were scanned three times at a scanning speed of 120 nm/min using a 1 mm pathlength quartz cuvette. Finally, the secondary structure contents were determined using the CDNN Program (Applied Photophysics Ltd., Surrey, UK).

### 2.11. UV Absorption Spectral Analysis

UV spectrum data of the glycosylated samples (0.2 mg/mL) were recorded from 200 to 320 nm at a wavelength interval of 0.5 nm, using a UV-vis spectrophotometer (UV-4802, Unico [Shanghai] Instrument Co., Ltd., Shanghai, China) at 25 °C. PBS was used as the blank.

### 2.12. Intrinsic and Extrinsic Fluorescence Analysis

The intrinsic and extrinsic fluorescence spectra of the glycosylated samples (0.2 mg/mL) were determined following the method described by Zhao et al. [15] using an F-4600 spectrofluorometer (Horiba Ltd., Kyoto, Japan). Prior to ANS fluorescence measurements, each sample (2.0 mL) was incubated with 25 μL of 8.0 mM ANS solution for 5 min. Intrinsic fluorescence emission data were collected between 300 and 400 nm at an excitation wavelength of 280 nm, while ANS fluorescence emission data were collected between 400 and 600 nm at an excitation wavelength of 380 nm. Both the excitation and emission slit widths were set at 5 nm.

### 2.13. Identification of Glycation Sites

Preparation of enzymatically digested peptides from glycosylated TM samples: first, samples with different glycosylation patterns were subjected to enzymatic digestion using trypsin at a substrate-to-enzyme ratio of 1:50 (*w*/*w*). The enzymatic digestion was performed overnight at 37 °C. The digested samples were then subjected to ultrafiltration using 10 kDa cutoff filters to remove undigested proteins, and the filtrate was collected. Subsequently, all samples were desalted using ZipTip C18 columns. The specific protocol is as follows: each sample was washed five times with pure acetonitrile washing solution, activated with a 50% acetonitrile aqueous solution (*v*/*v*), equilibrated with 0.1% formic acid aqueous solution (*v*/*v*), and then repeatedly aspirated and expelled 15–20 times for sample adsorption. After that, the ZipTip C18 columns were washed three times with 0.1% formic acid aqueous solution (*v*/*v*) for desalting. Gradient elution was performed using different elution solutions: 20 μL of 0.1% formic acid aqueous solution and 20% acetonitrile aqueous solution (*v*/*v*), 20 μL of 0.1% formic acid aqueous solution and 50% acetonitrile aqueous solution (*v*/*v*), and 20 μL of 0.1% formic acid aqueous solution and 70% acetonitrile aqueous solution (*v*/*v*). The eluates were collected in small vials, and 100 μL of 0.1% formic acid was added for subsequent HPLC-MS/MS analysis.

HPLC conditions: the chromatographic column used was a Hypersil gold C18 column (1.9 μm, 2.1 mm × 100 mm). The flow rate was set at 0.2 mL/min, the injection volume was 20 μL, and the column temperature was maintained at 40 °C. The mobile phases A and B comprised 0.1% formic acid–acetonitrile solution (*v*/*v*) and 0.1% formic acid–water solution (*v*/*v*), respectively. The adsorbed peptides were dynamically eluted and separated according to the following gradient program: 0–1 min, 5%A, and 95%B; 1–2.5 min, 5–10%A; 2.5–12.5 min, 10–25%A; 12.5–20 min, 25–52.5%A; 20–22 min, 52.5–90%A; 22–24 min, 90–5%A; 24–30 min, 5%A and 95%B.

MS/MS analysis parameters: the analysis was performed in positive ion mode using electrospray ionization (ESI+). The spray voltage was set at 3.6 kV, the capillary temperature was 300 °C, the sheath gas (N_2_) flow rate was 35 arb, and the auxiliary gas flow rate was 10 arb. The scan mode was set as full scan + data-dependent MS2 mode, with a scanning range of *m*/*z* 200–200, a resolution of 70,000, a normalized collision energy of 30, and a resolution of 17,500.

Database search and analysis: mass spectrometry data of the digested peptide segments from the glycosylated TM samples were searched against a protein database using Proteome Discoverer 1.4 software (Thermo-Fisher Scientific, Bremen, Germany). The parameters were set as follows: a peptide precursor ion tolerance of 10 ppm, MS/MS matching tolerance of 0.5 Da, allowance of up to 3 missed cleavage sites, high peptide confidence level, and trypsin was used as the protease. The fixed modification was carboxymethylation, and the variable modifications included oxidation, carbamylation, and deamidation.

### 2.14. Statistical Analysis

Experimental data were analyzed and plotted using SPSS 25.0 and Origin 2022b. Data were presented as mean ± standard deviation (n = 3). Statistical significance analysis was conducted using the ANOVA method, with *p* < 0.05 considered indicative of a statistically significant difference.

## 3. Results and Discussion

### 3.1. Analysis of the Degree of TM Glycosylation

#### 3.1.1. SDS-PAGE Analysis

**Figure 1:**
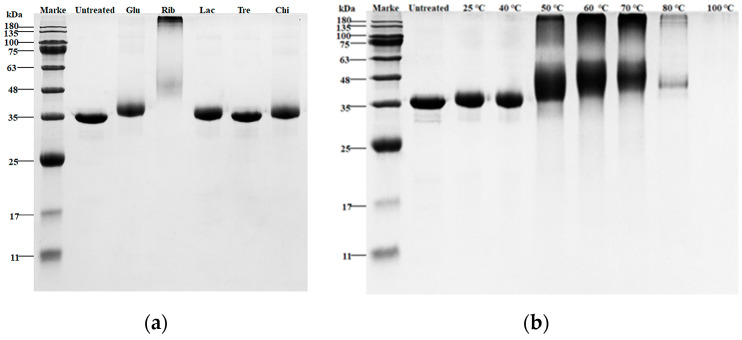
SDS-PAGE analysis of different TM glycosylation samples. (**a**) Glycation with different saccharides; (**b**) Glycation with different temperatures.

SDS-PAGE analysis was performed to analyze the molecular weight changes in the glycosylated TM samples under different types of sugars and temperatures. The results are shown in Figure 1. Compared with untreated TM, the bands of Glu, Rib, Lac, and Chi glycosylation samples with TM at 60 °C showed varying degrees of migration (Figure 1a). Among them, the Rib-TM glycosylation sample showed the most significant increase in molecular weight, accompanied by protein band diffusion and the formation of many large molecular glycosylation complexes near the separation gel wells. The Glu−TM and Lac-TM showed a lower increase in molecular weight. Notably, there was no significant difference in molecular weight between the Tre-TM glycosylation sample, Chi-TM glycosylation sample, and untreated TM. Rib is a pentose sugar and has the smallest molecular weight among the five different sugars, making it more prone to undergo glycosylation reactions with TM. On the other hand, Tre and Chi are non-reducing disaccharides and oligosaccharides, respectively, and cannot undergo glycosylation reactions with TM.

Furthermore, the effect of different reaction temperatures on the degree of TM glycosylation was investigated using the Rib−TM glycosylation reaction system. As shown in Figure 1b, under glycosylation reaction conditions below 40 °C, the Rib−TM glycosylation sample showed a slight increase in molecular weight compared with untreated TM. With an increase in glycosylation reaction temperature, the Rib-TM glycosylation sample exhibited a significant increase in molecular weight, accompanied by protein band diffusion and the formation of large molecular aggregates. It is worth noting that the Rib−TM glycosylation sample under glycosylation reaction conditions above 80 °C showed clear insolubility, and the Rib-TM glycosylation sample under glycosylation reaction conditions at 100 °C was hard to detect using protein bands in SDS-PAGE.

#### 3.1.2. Free Amino Group Analysis

**Figure 2:**
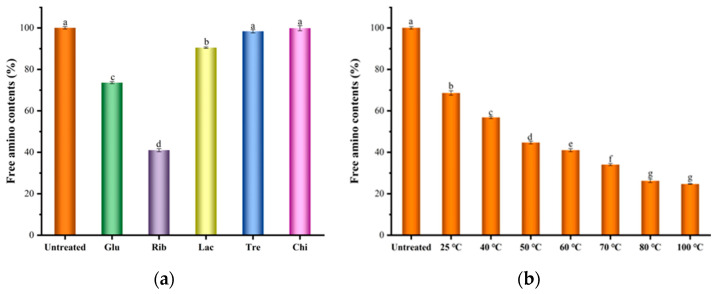
Analysis of free amino group content of different TM glycosylation samples. (**a**) Glycation with different saccharides; (**b**) Glycation with different temperatures. Different lowercase letters (a, b, c, d, e, f, and g) in column mean significant differences (*p* < 0.05) among different samples.

Glycation reactions are widely present in the thermal processing of food, and the glycation of proteins is mainly due to non-enzymatic reactions between free amino groups on the protein surface and sugar molecules. Therefore, the content of free amino groups in TM glycation samples can be used as an important indicator to evaluate the degree of TM glycation [1,7]. TM is rich in lysine residues (29 aa/284 aa), making it susceptible to glycation reactions with reducing sugars during heat treatment. As shown in Figure 2a, different types of sugar molecules have distinct effects on the content of free amino groups in TM. The order of free amino group content in TM is as follows: TM = Tre−TM = Chi−TM > Lac−TM > Glu−TM > Rib−TM, with a reduction of approximately 59.98% in Rib-TM. Lower content of free amino groups indicates a higher degree of glycation and more glycation sites. The significant decrease in the content of free amino groups further suggests that Rib can readily undergo glycation reactions with TM, whereas the non-reducing disaccharide Tre and oligosaccharide Chi hardly participate in glycation reactions with TM. This is consistent with the results from the SDS−PAGE analysis of TM glycation samples with different types of sugars. Nakamura et al. [8] also found a similar significant decrease in the content of free amino groups in the glycation study of TM from scallops (*Patinopecten yessoensis*) when using different types of sugars (glucose, maltose, and maltotriose). Zhang et al. [10] performed glycation reactions between TM from *Exopalaemon modestus* and sugars of different molecular weights (glucose, maltose, maltotriose, maltopentaose, or maltoheptaose) and observed a significant decrease in the content of free amino groups under different sugar systems. Within a certain range of molecular weights, smaller sugar molecules were found to more readily undergo glycation reactions with TM, primarily due to the influence of steric hindrance by sugar molecules. The effect of different reaction temperatures on the content of free amino groups in Rib-TM glycation samples is shown in Figure 2b. With increasing reaction temperature, the content of free amino groups in TM significantly decreases, indicating that higher temperatures promote glycation reactions. This result is consistent with the SDS-PAGE analysis mentioned above.

### 3.2. Effect of Different Glycosylation Treatments on TM IgG/IgE Binding Capacity

#### 3.2.1. WB Analysis

**Figure 3:**
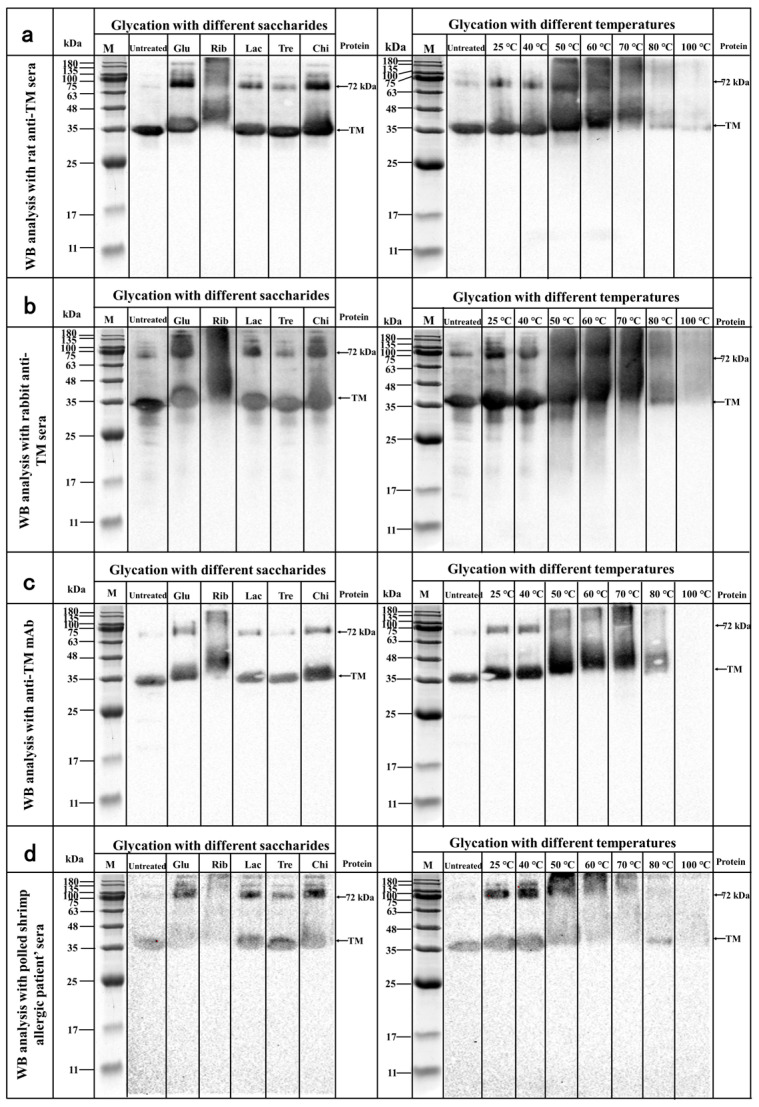
WB analysis of the effect of different glycosylation on the IgG/IgE binding capacity of TM. (**a**) TM rat WB analysis of glycosylated TMs; (**b**) TM rabbit polyclonal antisera WB analysis of glycosylated TMs; (**c**) mAb WB analysis of glycosylated TMs; (**d**) polled shrimp allergic patients’ sera WB analysis of glycosylated TMs.

The antibodies used to construct the PcAb and mAb sELISA methods and human sera from shrimp allergic patients were used to assess the effect of different glycosylation treatments on TM IgG/IgE binding capacity by WB analysis, and the results are shown in Figure 3. Based on the WB analysis results, it can be seen that different glycosylated TMs showed similar immunoblotting changes in the WB analysis results from the different antibodies. Compared with untreated TM, different types of glyco-TM glycosylated samples showed different degrees of change in the intensity of IgG/IgE immunoblotting in TM rat polyclonal antisera (Figure 2a), TM rabbit polyclonal antisera (Figure 3b), mAb (Figure 3c) and human serum from shrimp allergic patients (Figure 3d) with the magnitude of immunoblotting intensity as follows: TM = Tre−TM = Chi−TM = Lac−TM > Glu−TM > Rib−TM. In addition, the results of the IgG/IgE WB analysis of TM glycosylated samples at different temperatures showed that the IgG/IgE immunoblotting intensity of TM did not change significantly compared with TM at temperatures below 40 °C in the glycosylation reaction system and decreased significantly upon further increases in temperature. Notably, the trend of IgG/IgE immunoblotting intensity of different glycosylated TMs was consistent with their glycosylation degree, indicating that the IgG/IgE binding capacity of TMs decreased significantly with increasing glycosylation degree. Zhang et al. [14] analyzed the relationship between different molecular weight sugars (glucose, maltose, maltotriose, maltopentose, or maltheptose) and the maltose of *Hidrobacter* spp. (*Exopalaemon modestus*) by WB. Bai et al. [17] used arabinose, fructose, glucose, galactose, and mannose for glycosylation with scallop (*Chlamys nobilis*) TM and found that the WB analysis of glycosylated TMs showed a similar decrease in IgE blotting intensity. The intensity of IgG immunoblotting was significantly reduced in all glycosylated TM samples, with the lowest IgG immunoblotting intensity in the pentacosylated xylose-TM glycosylated samples. The increase in the IgG/IgE immunoblot intensity of a glycosylated TM indicates a significant decrease in its IgG/IgE binding capacity, which may be attributable to the structural alterations and modifications of TM caused by glycosylation, resulting in the masking or destruction of its IgG/IgE epitopes, making it less recognizable by IgG/IgE [7,14,17].

#### 3.2.2. Indirect ELISA Analysis

**Figure 4:**
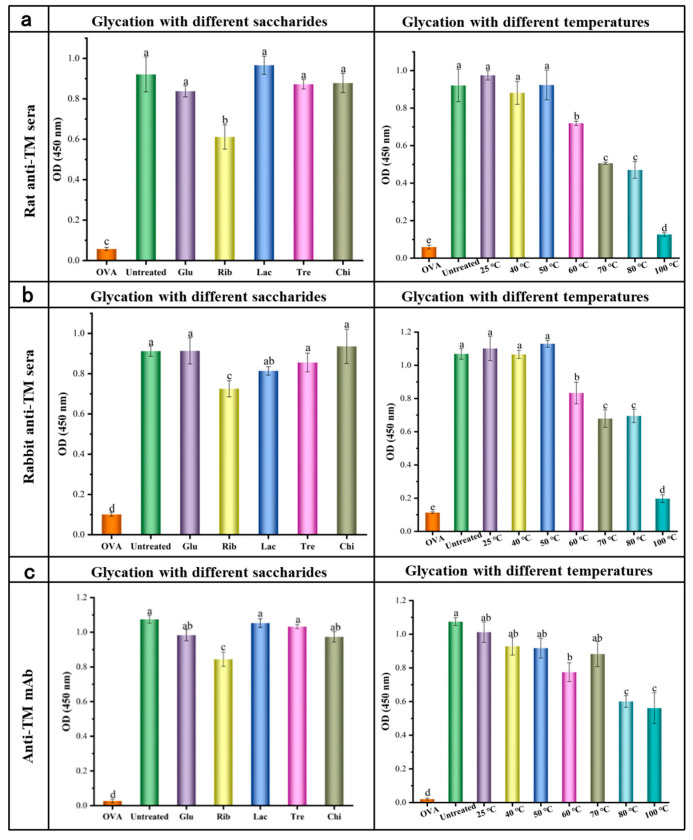
Effect of different glycosylation on the IgG binding capacity of TM by indirect ELISA analysis. (**a**) TM rat indirect ELISA analysis of glycosylated TMs; (**b**) TM rabbit polyclonal antisera indirect ELISA analysis of glycosylated TMs; (**c**) mAb indirect ELISA analysis of glycosylated TMs. Different lowercase letters (a, b, c, and d) in column mean significant differences (*p* < 0.05) among different samples.

**Figure 5:**
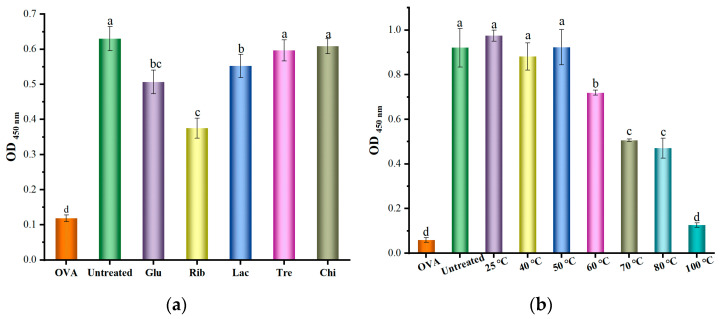
Effect of different glycosylation on the IgE binding capacity of TM by indirect ELISA analysis using polled shrimp allergic patients’ sera. (**a**) Glycation with different saccharides; (**b**) Glycation with different temperatures. Different lowercase letters (a, b, c, and d) in column mean significant differences (*p* < 0.05) among different samples.

The indirect ELISA analysis was further conducted using TM rat and rabbit polyclonal antisera, mAb, and human serum from shrimp allergic patients to evaluate the IgG/IgE binding ability to untreated and different glycosylated forms of TM, as shown in Figure 4 and Figure 5. Compared with untreated TM, there was no significant difference in the IgG binding ability of Glu, Lac, Tre, and Chi glycosylated TM (Figure 4a–c) (*p* ≥ 0.05), except for Rib−TM. However, there was a slight decrease in the IgE binding ability of Glu and Lac glycosylated TM (Figure 5). On the other hand, Rib glycosylated TM showed a significant reduction in IgG/IgE binding ability according to the indirect ELISA analysis with TM rat polyclonal antisera, TM rabbit polyclonal antisera, mAb, and human serum from shrimp allergic patients (*p* < 0.05), with reductions of approximately 27.3%, 25.8%, 50.6%, and 68.0% respectively.

Additionally, the IgG/IgE indirect ELISA analysis of Rib-TM under different temperature conditions revealed that there was no significant difference (*p* ≥ 0.05) in the IgG/IgE binding values of TM compared with untreated TM in the glycosylation reaction system below 50 °C (Figure 4 and Figure 5). However, upon further increases in reaction system temperature, there was a significant decrease (*p* < 0.05) in the IgG/IgE binding ability of TM. The trend in IgG/IgE variation for glycosylated TMs under different temperature reaction systems was also consistent with its degree of glycosylation; as the degree of glycosylation increased, there was a significant decrease in the IgG/IgE binding ability of TM. This is mainly due to the structural changes and modifications of TM antigenic epitopes caused by glycosylation, making it less recognizable by IgG/IgE antibodies [7,14,17]. It is worth noting that although Glu−TM, Lac−TM, and Rib-TM under the reaction system with a temperature below 50 °C undergo certain glycosylation reactions, their IgG binding ability did not show any significant change (*p* > 0.05) (Figure 4). This may be attributable to the significant structural changes in TM caused by heat treatment during the glycosylation reaction process, making it more recognizable by IgG, as well as the combined effect of epitope modification and destruction caused by glycosylation. In addition, in the indirect ELISA evaluation procedure, the excess antigen usually coats the 96-well ELISA plate, which might make it difficult to observe low levels of antigenic epitope modification and destruction in the indirect ELISA evaluation results [13].

### 3.3. Effect of Different Glycosylation Treatments on the Immunodetection Recovery of TM

**Figure 6:**
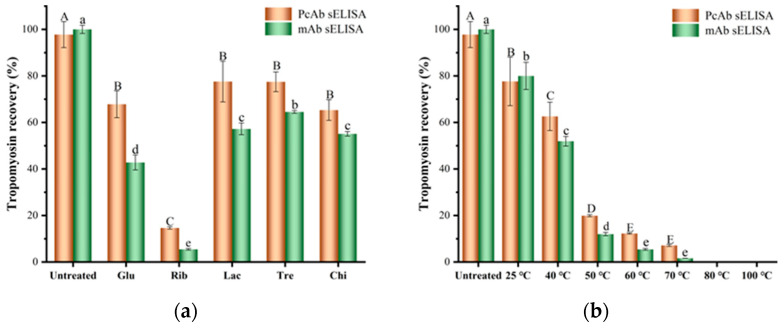
Effect of different glycosylation on the immunodetection recovery of TM via PcAb and mAb sELISA tool. (**a**) PcAb analysis; (**b**) mAb sELISA analysis. Different lowercase letters (a, b, c, d, and e) and uppercase letters (A, B, C, D, and E) in column mean significant differences (*p* < 0.05) among different samples.

The immunodetection recovery of TM with different glycosylation treatments was determined using the PcAb and mAb sELISA. As shown in Figure 6a, the detection recovery for glycosylated TMs with different types of sugars was significantly reduced compared with untreated TM (*p* < 0.05). The highest reduction was observed in Rib-TM, with decreases of 85.0% and 95.5% in PcAb and mAb sELISA analyses, respectively. This was followed by Glu-glycosylated TM (30.6% and 57.2%), Chi-glycosylated TM (33.2% and 44.9%), Lac-glycosylated TM (20.7% and 42.8%), and Tre-glycosylated TM (20.8% and 35.5%). As shown in Figure 6b, the detection recovery rate of Rib-glycosylated TM decreased rapidly and significantly with increasing temperature in different temperature systems (*p* < 0.05). In particular, Rib-glycosylated TM showed a nearly 100% reduction in the detection recovery rate in the glycosylation reaction system above 80 °C. The decreasing trend in immunodetection recovery of different glycosylated TMs was consistent with their degree of glycosylation. As the degree of glycosylation increased, the detection recovery rate for glycosylated TMs significantly decreased. This can be attributed mainly to the modification and destruction of TM antigenic epitopes caused by glycosylation treatment, leading to a significant reduction in the content of the “antibody–antigen–antibody” complex formation of TM in sELISA evaluation [5,13].

Furthermore, the immunodetection recovery of PcAb sELISA for the same glycosylated TM was significantly higher than that of mAb sELISA. This may be due to the fact that mAb only targets specific epitope regions on the protein molecule, while PcAb can undergo specific immune recognition with multiple epitope regions on the target protein molecule. As a result, the mAb sELISA system is more susceptible to structural changes in the target analyte, leading to a significant decrease in its detection accuracy [5,13]. It is worth noting that even though Glu, Lac, Chi, and Rib-glycosylated TM at glycosylation reaction temperatures below 50 °C show insignificant changes in IgG binding capacity, their PcAb and mAb sELISA immunodetection recovery were significantly reduced (*p* < 0.05). This may be attributable to the higher sensitivity of the sELISA detection system compared with the indirect ELISA evaluation system that only requires one antibody. Additionally, non-glycosylated Tre-glycosylated TM also exhibited a similar decrease in detection recovery rate, which may be due to the structural changes of TM during glycosylation and the non-covalent interaction between Tre and TM resulting in the masking of TM antigenic epitopes [18]. According to the sELISA procedure, at least two or more antibody binding sites are required in the target analyte to form the “antibody–antigen–antibody” sandwich complex. Structural changes and modifications in the target analyte can lead to occupancy or destruction of its IgG binding sites. The reduction in antibody binding sites and increased steric hindrance of adjacent antibody recognition sites make it more difficult for the detection antibody to bind to the target analyte, thus inhibiting the formation of the “antibody–antigen–antibody” complex and ultimately resulting in a significant underestimation of TM recovery rate [5,18,19]. These results indicate the important role of the number of antigen-binding sites in sELISA immunodetection.

### 3.4. Effect of Different Glycosylation Treatments on the In Vitro Digestibility of TM

**Figure 7:**
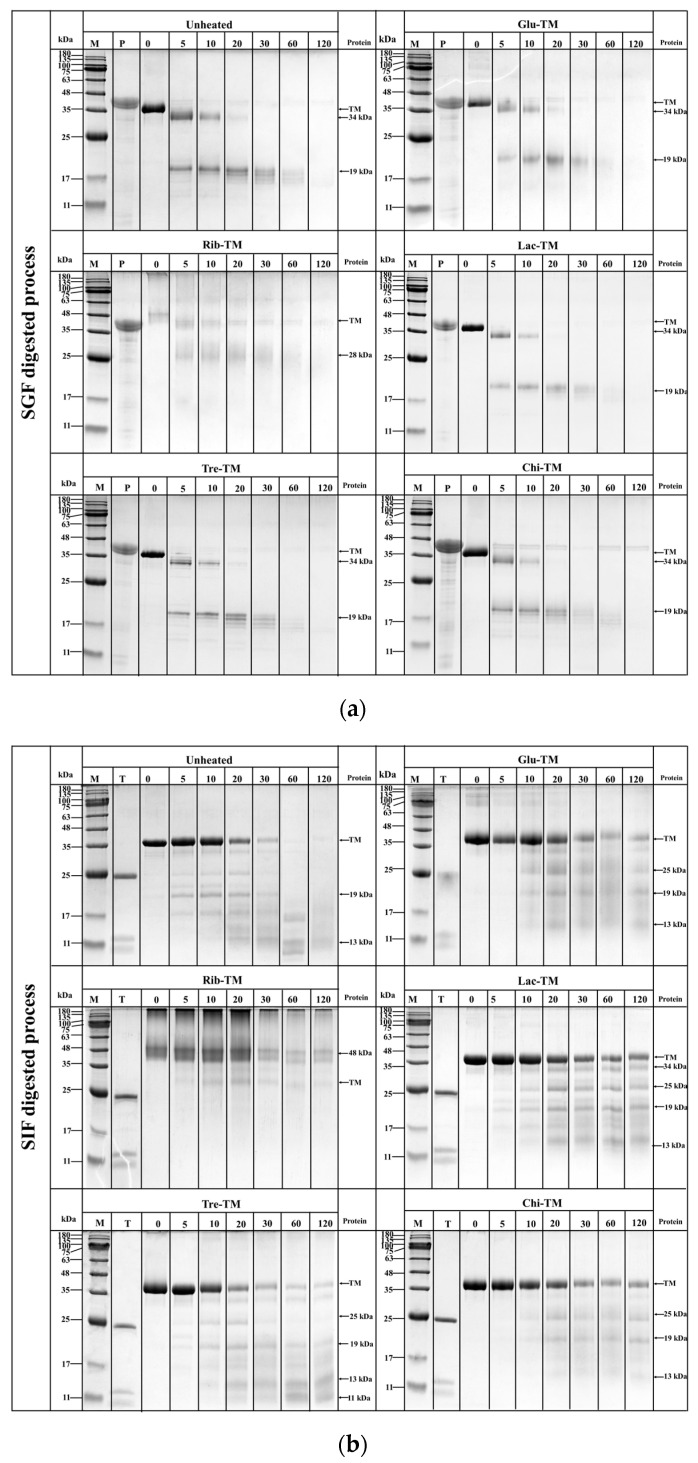

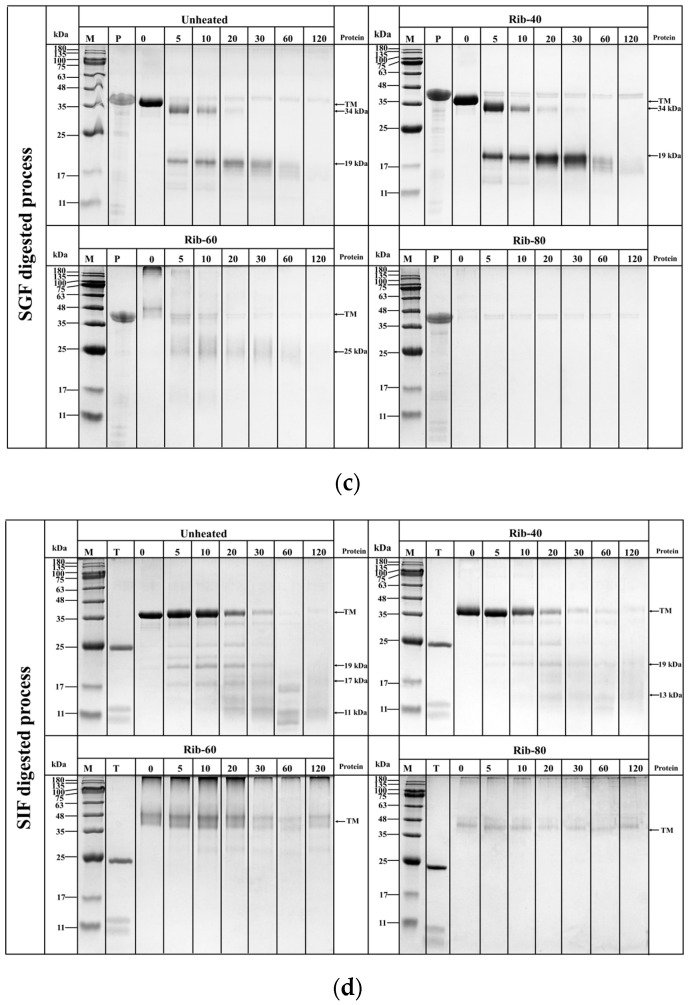
Effect of different glycosylation on the in vitro digestibility of TM via SDS-PAGE analysis. (**a**) SGF analysis of TM glycosylated with different sugars; (**b**) SIF analysis of TM glycosylated with different sugars; (**c**) SGF analysis of TM glycosylated with different temperatures; (**d**) SIF analysis of TM glycosylated with different temperatures.

The SDS-PAGE analysis of the in vitro digestion stability of TM influenced by different glycosylation treatments is shown in Figure 7. During the SGF digestion process, with increasing SGF digestion time, the band intensity of the untreated and differently glycosylated TM proteins gradually decreased, and different protein digestion fragment bands appeared. The SGF protein digestion fragments showed significant differences depending on the degree of TM glycosylation (Figure 7a,c). Among them, the untreated Tre and Chi-glycosylated TM proteins showed clear SGF protein digestion fragments near 19 kDa, while Glu and Lac-glycosylated TM proteins showed more dispersed SGF protein digestion fragments near 19 kDa. However, Rib-glycosylated TM proteins in different temperature reaction systems showed obvious dispersed SGF protein digestion fragments near 28 kDa. The differences in digestion fragments may be attributable to the shielding or disruption of the glycosylation TM gastric digestion sites. In addition, the digestion rates of TM with different glycosylation treatments were significantly different. The original TM protein bands could not be detected on SDS-PAGE within 20 min (untreated), 30 min (Glu), >120 min (Rib), 20 min (Lac), and 20 min (Chi) of SGF digestion time. These results indicate that glycosylation treatment significantly affects the gastric protease degradation resistance of TM, which may be attributable to structural changes caused by TM glycosylation that significantly reduce the gastric protease cleavage sites, resulting in a significant decrease in TM digestibility [20,21].

During the digestion process of untreated and differently glycosylated TMs in simulated intestinal fluid (SIF), the band intensity of both untreated and differently glycosylated TM proteins gradually decreased with increasing SIF digestion time (Figure 7b,d). It is worth noting that the rate of trypsin degradation differed among the differently glycosylated TM proteins, with untreated TM being completely degraded within 60 min of SIF digestion time, while the differently glycosylated TM proteins could still be detected on SDS-PAGE even after 120 min of simulated gastric fluid (SGF) digestion time. Additionally, abundant SIF protein digestion fragments appeared below 25 kDa molecular weight for the differently glycosylated TM proteins, and the SIF protein digestion fragments of untreated TM and differently glycosylated TMs also showed significant differences on the SDS-PAGE gels, especially for the SIF protein digestion fragments of Rib-TM. Similar observations of significantly reduced digestion were found in the in vitro simulated digestion experiments of glycosylated *Exopalaemon modestus* TM [20], *Scophthalmus maximus* parvalbumin [22], soy protein [23], and milk protein [24]. This is mainly attributed to the significant impact of glycosylation on the proteolytic degradation resistance of TM. Glycosylation treatment leads to significant changes and modifications in the structure of TM, resulting in the shielding or disruption of trypsin cleavage sites, thereby making TM less susceptible to trypsin digestion and degradation [20,21]. It is worth noting that TM without glycosylation (Tre and Chi) also exhibited a similar significant reduction in the in vitro digestion, which may be due to the presence of multiple trypsin cleavage sites in TM (Lys: 29 aa/284 aa; Arg: 21 aa/284), making it more susceptible to structural changes. Furthermore, although the non-reducing sugars Tre and Chi did not undergo covalent glycosylation modification with TM, they can still interact with proteins through non-covalent interactions such as hydrogen bonding and hydrophobic interactions due to the presence of multiple active groups within their sugar molecules [25,26], thereby masking or shielding the trypsin cleavage sites of TM and hindering its degradation by trypsin.

### 3.5. Effect of Different Glycosylation Treatments on the Structure of TM

#### 3.5.1. CD Analysis

**Figure 8:**
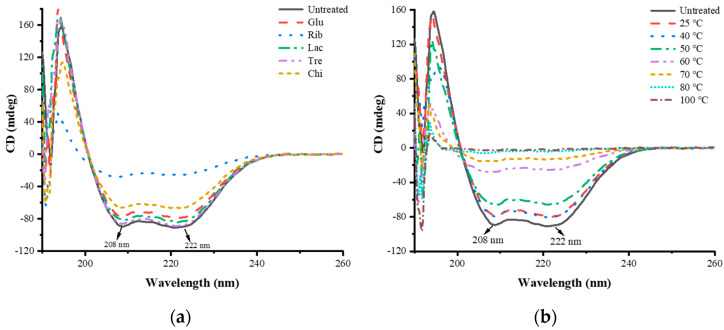
CD spectral analysis of different TM glycosylation samples. (**a**) TM glycosylated with different sugars; (**b**) TM glycosylated with different temperatures.

The impact of different glycosylation treatments on the secondary structure of TM can be understood through the analysis of CD spectra. The CD spectral analysis results of TM with different glycosylation treatments are shown in Figure 8. The CD spectrum of untreated TM exhibits a positive peak at 192 nm and two distinct negative peaks at 208 and 222 nm, which are typical features of protein secondary structure α−helix. The negative peak at 216 nm and the positive peak at 190–200 nm are characteristic absorption peaks of protein secondary structure β−sheet [27,28]. In addition, the CD spectrum curve shows that the CD signal value at 222 nm is lower than the CD signal value at 208 nm, which is a typical feature of the TM α−helix secondary structure [27,29]. After different glycosylation treatments, compared with the original CD spectrum of TM, the intensity of the negative peaks at 208 nm and 222 nm in the CD spectra of glycosylated TMs at different sugars and temperatures decreases to varying degrees. Among them, Rib-TM shows the most significant decrease, and the peak intensity gradually weakens with increasing temperature. Rib-glycosylated TM above 80 °C has no obvious negative peak near 208 nm and 222 nm. This indicates that glycosylation treatment significantly rearranges the secondary structure of TM, stretching and unwinding the α−helical structure into an irregular coil and β−turn structures, making the TM secondary structure more disordered. Among the different sugar-glycosylated TMs, the structural change is most pronounced in Rib-glycosylated TM, with the lowest α−helical structure content, followed by Chi−TM, Glu−TM, Lac−TM, and Tre−TM. Zhang et al. [14] used different functional oligosaccharides (oligogalactose, oligomannose, oligofructose, and maltopentaose) to glycosylate TM from *Exopalaemon modestus*, and CD spectrum analysis also revealed a significant decrease in α-helical structure content accompanied by a marked increase in irregular coil structure. Fu et al. [9] synthesized glycosylated TMs from *Penaeus chinensis* using ribose, oligogalactose, and chitooligosaccharides, and CD spectrum analysis showed a significant decrease in α-helical structure content accompanied by a significant increase in β−fold content. They also found that the maintenance of α−helical structure content plays an important role in the sensitivity of TM. In addition, untreated TMs (Tre and Chi) also showed a significant decrease in α−helical structure content, especially Chi, which may be due to the active groups of non-reducing sugars Tre and Chi being able to interact with the protein through non-covalent interactions such as hydrogen bonding and hydrophobic interactions [25,26], leading to the disruption of hydrogen bonding interactions that maintain TM α−helical structure. The above analysis of secondary structure changes indicates that glycosylation treatment results in significant changes in TM secondary structure, which may be the main factors inducing its IgG/IgE binding ability, immunodetection recovery, and gastrointestinal digestibility reduction.

#### 3.5.2. UV Spectral Analysis

**Figure 9:**
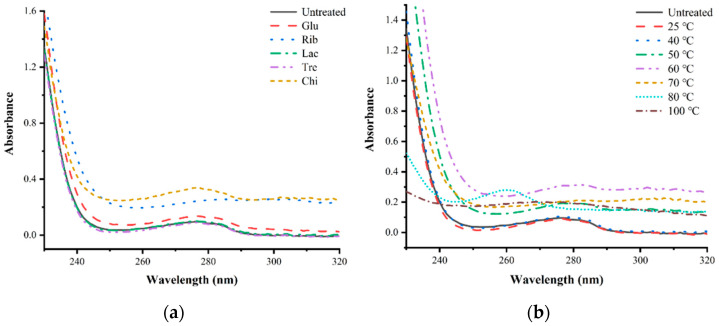
UV spectral analysis of different TM glycosylation samples. (**a**) TM glycosylated with different sugars; (**b**) TM glycosylated with different temperatures.

The results of the UV absorption spectral analysis of different glycosylated TMs are shown in Figure 9. Based on the UV absorption spectra, it can be seen that the maximum UV absorption peak for untreated TM is around 278 nm, and the Rib, Chi, and Glu glycosylated TMs show a clear increase in intensity corresponding to the maximum absorption peak of the UV spectrum; however, the UV absorption spectra of the Lac and Tre glycosylated TMs show no significant changes compared with untreated TM (Figure 9a). In addition, the analysis of the UV spectra of Rib−TM at different temperatures revealed that the intensity values corresponding to the maximum absorption peaks of the UV spectra of TM were not significantly different below 40 °C in the glycosylation reaction compared with untreated TM, and the intensity corresponding to the maximum absorption peaks of the UV spectra of Rib−TM increased significantly with increasing temperature (Figure 9b). The increase in the intensity of the maximum absorption peak of the UV spectrum of glycosylated TMs indicated that the microenvironment of amino acid residues within TM was significantly altered due to significant changes in the tertiary structure of TM as a result of the glycosylation treatment [15,29], which was also consistent with the results from the subsequent internal and external fluorescence spectroscopy.

#### 3.5.3. Intrinsic Fluorescence Spectral Analysis

**Figure 10:**
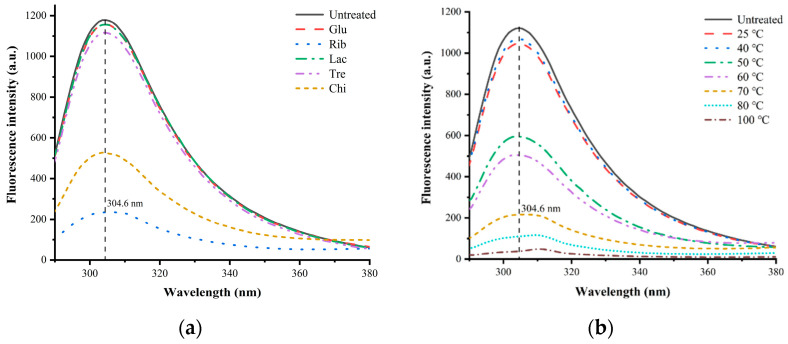
Intrinsic fluorescence spectral analysis of different TM glycosylation samples. (**a**) TM glycosylated with different sugars; (**b**) TM glycosylated with different temperatures.

The analysis of the intrinsic fluorescence spectra of TM with different glycosylation treatments is shown in Figure 10. From the analysis of intrinsic fluorescence spectra, it can be observed that the fluorescence emission wavelength (λ_max_) corresponding to the maximum absorption peak of the intrinsic fluorescence spectra of untreated TM is around 305.2 nm. With the application of glycosylation treatments, the maximum fluorescence intensity at λ_max_ of glycosylated TMs show varying degrees of increase, but there is no significant shift in λ_max_. Among them, the fluorescence intensity corresponding to λ_max_ of Rib−TM decreases most significantly, followed by Chi−TM, Tre−TM, Glu−TM, and Lac−TM samples (Figure 10a). In addition, the analysis of intrinsic fluorescence spectra of Rib-glycosylated TM under different temperature conditions found that with the increase in glycosylation reaction temperature, the fluorescence intensity values corresponding to λ_max_ of Rib-glycosylated TM gradually decrease compared with untreated TM (Figure 10b). The study shows that TM has a linear double helical structure feature, and in its natural state, the amino acids of TM are exposed to a polar environment, including hydrophobic amino acids that can produce fluorescence, such as Tyr residues. Glycosylation treatment results in covalent modification of the free amino groups of TM molecules with sugar molecules, leading to the hydrophobic aggregation of TM, and the fluorescence emission group Tyr residues are masked or shielded, ultimately resulting in a significant decrease in the intrinsic fluorescence intensity [30,31]. Lv et al. [32] used different levels of xylose to glycosylate TM with *Metapenaeus ensis* and found a similar significant reduction in endogenous fluorescence intensity using endogenous fluorescence spectroscopy. In addition, similar significant reductions in endogenous fluorescence intensity were also found for TM treated with different levels of acetone–aldehyde glycosylation, while indirect ELISA analysis revealed a significant reduction in the IgE binding capacity of acetone–aldehyde glycosylated TMs [33]. Notably, TM without glycosylation (Chi) also showed a significant reduction in endogenous fluorescence, which may be attributable to the fact that the reactive group of the non-reducing Chi sugar molecule can interact non-covalently with the protein [25,26], resulting in the masking or obscuring of the fluorescence emitting group Tyr, which ultimately leads to a significant reduction in the endogenous fluorescence intensity of TM. The above results suggest that glycosylation treatment resulted in significant changes in the tertiary structure of TM, which may be a major factor contributing to its reduced IgG/IgE binding capacity, immunodetection recovery, and gastrointestinal digestibility.

#### 3.5.4. Extrinsic Fluorescence Spectral Analysis

**Figure 11:**
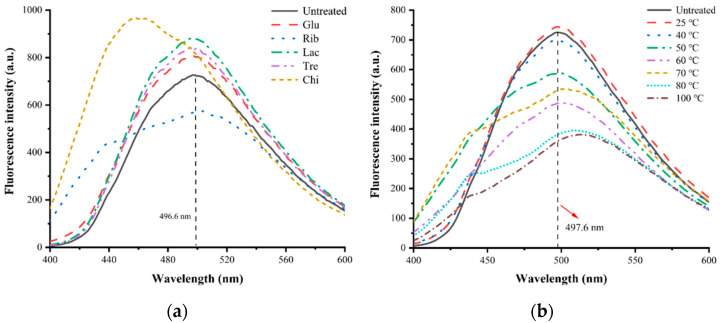
Extrinsic fluorescence spectral analysis of different TM glycosylation samples. (**a**) TM glycosylated with different sugars; (**b**) TM glycosylated with different temperatures.

The different glycosylation treatments of TM were further analyzed using ANS fluorescence spectra; the results are shown in Figure 11. Based on the extrinsic fluorescence spectrum analysis it can be seen that the maximum absorption peak of untreated ANS extrinsic fluorescence spectrum corresponds to the λ_max_ around 496.6 nm. With the application of different glycosylation treatments, the ANS fluorescence spectra of glycosylated TMs changed significantly, with the exception of the Rib glycosylated TM, the ANS fluorescence intensity of the Glu, Lac, Tre, and Chi glycosylated TM showed a significant increase. The extrinsic fluorescence spectra of Rib-TM at different temperature regimes revealed that the ANS fluorescence intensity gradually decreased with the increasing temperature of the glycosylation reaction, accompanied by a significant redshift. The above results indicate that mild glycosylation causes significant changes in the tertiary structure of TM, which can significantly increase the surface hydrophobicity of TM; as the degree of glycosylation increases, TM appears hydrophobic aggregation, eventually leading to a significant decrease in its ANS fluorescence intensity, further demonstrating that significant structural changes occur in glycosylation–treated TM [34]. This is also consistent with the results from the UV and extrinsic fluorescence spectroscopy analyses described above. These structural changes may result in the disruption or masking of TM epitopes and gastrointestinal enzyme digestion sites, thus ultimately leading to a significant reduction in IgG/IgE binding capacity, immunodetection recovery, and in vitro digestibility [7,35].

### 3.6. Modification of Specific TM Amino Acids via Glycosylation

**Figure 12:**
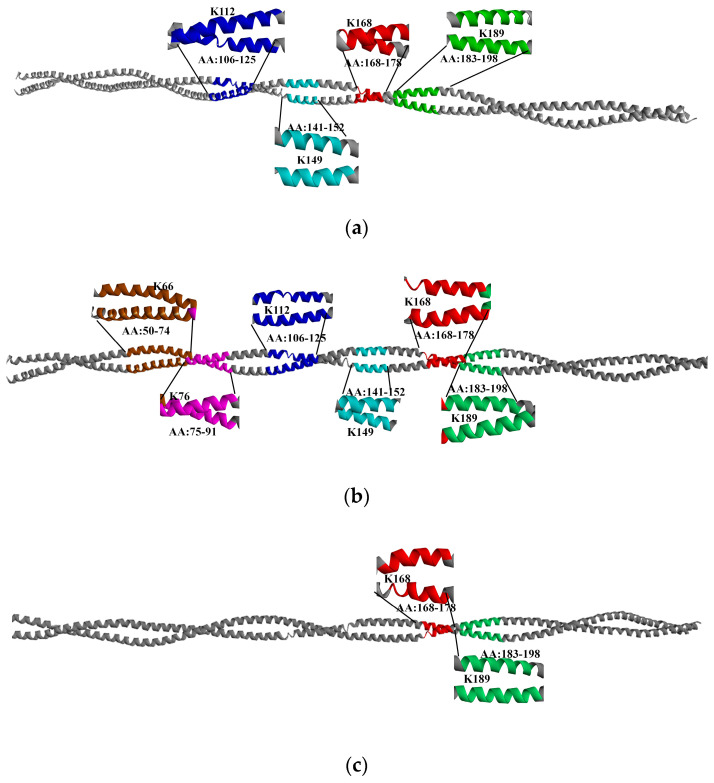
The 3D structure demonstration of glycosylation sites of TM. (**a**) Glu-TM; (**b**) Rib-TM; (**c**) Lac-TM.

To understand the mechanism of the effect of glycosylation treatment on the IgG/IgE binding capacity, immunodetection, and in vitro digestibility of TM, the TM glycosylation sites with different glycosylation reactions were further identified using Q Exactive mass spectrometry. As shown in Table 1, compared with untreated TM, 4 (Glu−TM), 8 (Rib−TM), 2 (Lac−TM), 0 (Tre−TM), and 0 (Chi−TM) glycosylation-modified peptides were identified for different glycosylation-treated TM, where Glu−TM was identified with four different glycosylation sites (K112, K149, K168, and K189), Rib-TM was identified to contain six different glycosylation sites (K66, K76, K112, K149, K168, and K189) and Lac−TM contained two different glycosylation sites (K168 and K189), where it can be observed that K is the specific amino acid modified by the TM glycosylation reaction. According to the protein sequence of shrimp TM (*Litopenaeus vannamei*) (NCBI ID: ACB38288), it is known that TM contains multiple Lys(K) (29 aa/284 aa), which are susceptible to glycosylation reactions with reducing sugars [7,20]. In addition, different glycosylated TMs (Glu−TM, Rib−TM, and Chi−TM) were identified with glycosylation fragments and glycosylation sites localized to the TM 3D structure, and the results are shown in Figure 12.

Further analysis of the effects of different glycosylation sites on the IgE binding epitopes of TM reveals that Glu-TM has two IgE binding epitopes that are glycosylated, namely E5b: 142–162 and E5c: 157–183. Rib–TM has three glycosylated IgE binding epitopes (E3: 61–81, E5b: 142–162, and E5c: 157–183). One glycosylated modification site (E5c: 157~183) was identified in Glu-TM. Based on these results, it can be concluded that glycosylation leads to differential glycosylation of TM epitopes, resulting in the destruction or masking of these epitopes. As a result, the IgG/IgE ability of TM, immunodetection, and in vitro digestibility are significantly reduced [17,35]. Han et al. [35] used different reducing sugars (ribose, arabinose, lactose, glucose, and maltose) to perform glycosylation reactions with TM from *Scylla paramamosain*. Mass spectrometry analysis revealed three glycosylation sites (K112, R125, R133) within TM IgE binding epitopes, which may be the main factors leading to a decrease in IgE binding ability. Studies have reported that glycosylation modification of *Exopalaemon* modestus TM with reducing sugars of different molecular weights disrupts or masks multiple key amino acid residues (Lys) within these TM epitopes, resulting in a significant decrease in IgE binding ability and ultimately reducing the allergenicity of different glycosylated TMs. Further analysis of the impact of different glycosylation sites on the binding epitopes of TM IgE reveals that Glu-TM contains two IgE binding epitopes that are glycosylated, namely E5b: 142–162 and E5c: 157–183. Additionally, three IgE binding epitopes (E3: 61–81, E5b: 142–162, and E5c: 157–183) in Rib-TM were found to be modified. Furthermore, one glycosylated modification site (E5c: 157–183) was identified in Glu–TM. These results suggest that glycosylation modification leads to varying degrees of glycosylation of TM epitopes, causing their dilution or masking, ultimately resulting in a significant decrease in its IgG/IgE binding capacity and immunodetection recovery [17,35]. In a study conducted by Han et al. [35], various reducing sugars (ribose, arabinose, lactose, glucose, and maltose) were used for glycosylation reaction with TM from Crab (*Scylla paramamosain*). Mass spectrometry analysis revealed that three glycosylation sites (K112, R125, R133) were discovered in TM IgE epitopes, which may be the main factors contributing to decreased IgE binding ability. Another study reported that glycosylation modifications by different molecular weight reducing sugars disrupted or masked multiple key lysine residues in the TM epitopes of *Exopalaemon modestus*, leading to a significant decrease in its IgE binding capacity and ultimately weakening the allergenicity of different glycosylated TMs [37]. Additionally, Bai et al. [17] performed glycosylation reactions using arabinose, xylose, glucose, mannose, and lactose with TM from noble scallop (*Chlamys nobilis*). Through indirect ELISA, basophil activation assay, and shotgun proteomics analysis, it was observed that six IgE binding epitopes of TM were modified by glycosylation, resulting in a significant decrease in its allergenicity.

## 4. Conclusions

In conclusion, the IgG/IgE binding ability, immunodetection, in vitro digestibility, and structural changes in TM glycosylated with different types of sugars (Glu, Rib, Lac, Tre, and Chi) and various glycosylation temperatures were investigated. The IgG/IgE binding ability of glycosylated TM, immunodetection recovery, and in vitro digestibility all show a significant decrease after glycosylation treatment. The extent of reduction depends on the degree of glycosylation, with Rib (pentose sugar) glycosylated TM exhibiting the greatest reduction, followed by Glu, Lac, Tre, and Chi. Additionally, as the glycosylation temperature increases, a decrease in binding ability to IgG/IgE, immunodetection recovery rate, and in vitro digestibility becomes more pronounced. Based on CD, UV, and intrinsic/extrinsic fluorescence spectroscopy analyses, significant structural changes are observed in glycosylated TM. The secondary structure undergoes rearrangement, with the typical linear α-helical structure of TM being disrupted and gradually transformed into β-turns and irregular coils during heat treatment. The three-dimensional structure becomes twisted and folded, and the surface hydrophobicity increases significantly, even leading to hydrophobic aggregation. Glycosylation sites are identified using HPLC-MS/MS, with three epitopes (E3: 61–81, E5b: 142–162, and E5c: 157–183) of TM found to be susceptible to glycosylation modification. It is worth noting that Tre-TM and Chi-TM, which did not undergo glycosylation modification, also exhibit a significant decrease in the immunodetection recovery rate. This suggests that different sugar molecules may not only lead to a loss of IgG binding sites on TM through structural disruption and epitope modification but also produce non-covalent interactions between sugar molecules and the target protein, resulting in the masking or shielding of antigenic epitopes on the target protein, thereby reducing the recovery rate of the target protein in immunodetection.

## Figures and Tables

**Table 1:** Glycosylation modification sites of TM glycosylated with different sugars.

Samples	Modification Sites	Identified Peptide	Location	IgE Epitopes [36]
Glu-TM	K112	LNTATTKLAEASQAADESER	106–125	-
	K149	MDALENQLKEAR	141–152	E5b: 142~162
	K168	KLAMVEADLER	168–178	E5c: 157~183
	K189	AETGESKIVELEEELR	183–198	-
Rib-TM	K66	MQQLENDLDQVQESLLKANIQLVEK	50–74	E3: 61~81
	K76	DKALSNAEGEVAALNR	75–90	E3: 61~81
	K76	DKALSNAEGEVAALNRR	75–91	E3: 61~81
	K112	LNTATTKLAEASQAADESER	106–125	-
	K149	MDALENQLKEAR	141–152	E5b: 142~162
	K168	KLAMVEADLER	168–178	E5c: 157~183
	K168	KLAMVEADLERAEER	168–182	E5c: 157~183
	K189	AETGESKIVELEEELR	183–198	-
Lac-TM	K168	KLAMVEADLER	168–178	E5c: 157~183
	K189	AETGESKIVELEEELR	183–198	-
Tre-TM	-	-	-	
Chi-TM	-	-	-	

## Data Availability

Data presented in this study are available on request from the corresponding author.

## Supplementary Materials

The following supporting information can be downloaded at: https://www.mdpi.com/article/10.3390/foods12163049/s1, Table S1. Serological characterization of shrimp-allergic patients.
