# Supplementary material for: Insights into the Mechanism Underlying the Influence of Glycation with Different Saccharides and Temperatures on the IgG/IgE Binding Ability, Immunodetection, In Vitro Digestibility of Shrimp (Litopenaeus vannamei) Tropomyosin
Source: Foods. 2023 Aug 14;12(16):3049. doi: 10.3390/foods12163049 (PMC10453262; doi:10.3390/foods12163049)
Supplement: Supplementary file 1 [file foods-12-03049-s001.zip › foods-2525166-supplementary.pdf]

Table S1. Serological characterization of shrimp-allergic patients.

**Serum No.**	**Shrimp sIgE (kU/L)**
P1	20.7
P2	18.1
P3	16.2
P4	15.1
P5	27.4
P6	32.2
N1	⩽0.35
N2	⩽0.35
